# Clinical electrophysiology in the Netherlands: where do we stand?

**DOI:** 10.1007/s12471-017-1030-z

**Published:** 2017-08-02

**Authors:** J. R. de Groot

**Affiliations:** Heart Center, Department of Cardiology, Academic Medical Center, University of Amsterdam, Amsterdam, The Netherlands

For more than a century, Dutch scientists and clinicians have contributed importantly to the understanding of cardiac electrophysiology and to the diagnosis and treatment of cardiac rhythm disturbances. The first example supporting this statement that comes to mind is the development of a string galvanometer and the registration of the human electrocardiogram by Willem Einthoven in 1901 [1]. Almost 100 years after Einthoven’s Nobel prize in 1924 the ECG is still the most important tool for virtually everyone working in the field of cardiology. The first description of the total excitation of the human heart, recorded with hundreds of intramural electrodes implanted in an explanted, Langendorff perfused human heart stems from Dutch soil [2]. Similarly, the cradle of invasive electrophysiology stood in the Wilhelmina Gasthuis, where Wellens and associates, simultaneously with Coumel’s group in Paris, developed programmed stimulation that has become a standard approach to discern arrhythmia mechanisms [3]. In pacing, Dutch investigators played a central role, for example in a large study on appropriate pacemaker follow-up, as well as in the recent first-in-man studies of completely leadless pacemakers [4–6]. Hence, we look back at a glorious past, but what is the position of clinical electrophysiology in the Netherlands today, and what should we expect from the future?

This issue of the Netherlands Heart Journal is focused on electrophysiology and aims at answering that very question. Having left the pioneering era behind us, the field of clinical electrophysiology has grown over the past years, both worldwide and in the Netherlands. In 2016, our country saw ≈ 10,000 pacemaker implantations, more than 5000 ICD implantations and 8750 catheter ablations, of which 4000 for atrial fibrillation (AF).

With the increase in the number of AF ablations, a shift in the characteristics of patients undergoing these procedures is to be expected. Teunissen et al. describe how patient and procedure characteristics of pulmonary vein isolation for AF changed in 975 procedures performed in a single centre between 2005 and 2015 [7]. They show that over this period the percentage of patients with persistent AF doubled, as did the percentage of patients with a CHADS-VASc score ≥ 2. The average age increased by 7 years, whereas the duration of AF symptoms decreased from 7 to 4 years. Meanwhile, procedure time, radiation time and complications, pulmonary vein stenosis and vascular complications in particular, drastically decreased. However, and employing a systematic follow-up protocol, the 1‑year success rate remained constant over these 10 years, at approximately 55%. One interpretation may be that, although the procedure has matured as was evident from a shorter procedure time and fewer complications, an expected concomitant increase in efficacy was counterbalanced by a patient population with a lesser prognosis because of more advanced AF and more comorbidities. As patients were referred for ablation after a shorter history of AF, awareness of the arrhythmia and invasive treatment options seems to have increased. In the early stage of the disease, episodes of AF may be paroxysmal and go unnoticed, yet carry an increased risk for stroke in particular. Hence the need for documentation of the arrhythmia is obvious. Verbiest-van Gurp et al. describe how cardiologists in the Netherlands use available technology to detect AF [8]. Ninety hospitals with a cardiology department (outpatient clinics were excluded) were selected. One cardiologist per centre, assumed to represent that clinic, was approached for an online questionnaire (response 53%). Questions on which monitoring strategy is indicated were guided by six clinical vignettes. Particularly with signs or symptoms of AF, virtually all cardiologists would initiate further investigation, commonly escalating from a 12-lead EGC, to more prolonged monitoring or patient-activated monitoring devices as a secondary approach. Aside from rhythm monitoring, 98% of cardiologists would perform an echocardiogram in the workup for the diagnosis. Hence, cardiologists are, contrary to what is advised to general practitioners in the Dutch College of General Practitioners (NHG) guideline, well aware of the fact that all AF patients need a cardiological workup. A thorough understanding of the clinical conditions, after all, is imperative for patient-tailored therapy and prevention of stroke in particular. Therefore, Pisters et al. assessed the prescription patterns and drug safety of rivaroxaban in the Dutch patients included in the Xarelto for the prevention of stroke in patients with atrial fibrillation (XANTUS) registry [9]. XANTUS is a European prospective, observational registry on rivaroxaban use in patients with non-valvular AF. The Dutch patients were younger and less often had permanent AF than the patients in the overall XANTUS cohort. Nineteen patients experienced major bleeding (>40% gastrointestinal bleeding, 20% intracranial haemorrhage), corresponding to a rate of 2.4/100 patient years. Interestingly, label-discordant dose reduction was observed in 8.3% of patients whereas, for example, insurance data from the US indicate a far higher percentage [10]. Taking both observations into consideration, a prospective registry may not represent real life as well as real life does. A similar discrepancy may exist with regard to the patients with an indication for an implantable cardioverter defibrillator (ICD) for primary prevention of sudden death, which may be different in the randomised studies that shaped our guidelines than in the real world. Van Barreveld et al. describe the design and baseline patient characteristics of the nationwide prospective Dutch outcome in ICD therapy (DO-IT) registry [11]. They mention several potential causes of this discrepancy, including improvement of primary revascularisation for acute myocardial infarction and better uptake of heart failure treatment. Subsequently, they included 1468 patients from all 28 ICD implanting centres in the country, and collected data on demographics and normal follow-up. The primary outcomes of the study are death and appropriate ICD therapy for ventricular tachycardia or fibrillation. Interestingly, the demographics of the study cohort compare fairly well with the MADIT-II and SCD-HeFT cohorts, with a slightly higher left ventricular ejection fraction (mean 26 vs. 23% (MADIT-II) or median 27 vs. 24% (SCD-HeFT)) and a lower percentage of patients with NYHA class 3 heart failure symptoms (22 vs 30 and 30%). The fear that ICDs are mainly implanted in patients in whom the left ventricular ejection fraction is 35% or just below seems therefore unjustified. Future analysis of DO-IT will include a prediction model for the primary outcome and an economic evaluation of both current practice and the prediction model.

Device therapy can be life-saving, but is also associated with complications related to lead failure and pulse generator exchanges, which in turn may cause infection. Battery longevity therefore forms an important determinant of device safety. De Vries et al. investigated the temporal trends in service time of pacemakers between 1984 and 2006, making use of a national device database [12]. Almost 97,000 patients were grouped in strata of implantation date with 7 years of follow-up or until explantation. Only approximately 50% of devices were explanted because of normal ‘end of life’. Of the other devices, 19% were replaced or removed following device failure or complications. Complication and failure rates did not improve over the more than 20-year study period, and the lifetime of the device actually decreased. This observation provokes contemplation on the balance between the plethora of available algorithms and monitoring functions that all consume energy, in relation to the device lifetime and the implications device exchange has for the patient.

The five original contributions to this issue of the Netherlands Heart Journal show that clinical electrophysiology is being practised and studied at a high level in the Netherlands. All studies were generally well designed and carried out with care. Importantly, relevant and complete follow-up of patient outcomes is emphasised by the investigators. This will truly improve understanding of the potential and limitations of different therapies, also beyond the electrophysiological community. With these contributions, being representative for contemporary Dutch electrophysiology, the future can be looked forward to with confidence.
